# Potential‐Dependent Morphology of Copper Catalysts During CO_2_ Electroreduction Revealed by In Situ Atomic Force Microscopy

**DOI:** 10.1002/anie.202010449

**Published:** 2020-12-01

**Authors:** Georg H. Simon, Christopher S. Kley, Beatriz Roldan Cuenya

**Affiliations:** ^1^ Department of Interface Science Fritz Haber Institute of the Max Planck Society 14195 Berlin Germany; ^2^ Young Investigator Group Nanoscale Operando CO_2_ Photo-Electrocatalysis Helmholtz-Zentrum Berlin für Materialien und Energie GmbH 14109 Berlin Germany

**Keywords:** CO_2_ electroreduction, copper electrocatalysis, in situ scanning probe microscopy, nanoelectrochemistry, surface chemistry

## Abstract

Electrochemical AFM is a powerful tool for the real‐space characterization of catalysts under realistic electrochemical CO_2_ reduction (CO_2_RR) conditions. The evolution of structural features ranging from the micrometer to the atomic scale could be resolved during CO_2_RR. Using Cu(100) as model surface, distinct nanoscale surface morphologies and their potential‐dependent transformations from granular to smoothly curved mound‐pit surfaces or structures with rectangular terraces are revealed during CO_2_RR in 0.1 m KHCO_3_. The density of undercoordinated copper sites during CO_2_RR is shown to increase with decreasing potential. In situ atomic‐scale imaging reveals specific adsorption occurring at distinct cathodic potentials impacting the observed catalyst structure. These results show the complex interrelation of the morphology, structure, defect density, applied potential, and electrolyte in copper CO_2_RR catalysts.

## Graphical abstract

Copper catalyst for electrochemical CO_2_ conversion has been unraveled in situ down to the atomic scale using electrochemical atomic force microscopy under highly gas‐evolving reaction conditions. Oxidized copper surfaces enter different morphological regimes in cathodic potential ranges down to −1.1 V_RHE_ coupled with surface reconstructions and varying density of undercoordinated reaction sites.

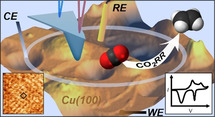

## Introduction

The electrochemical reduction of CO_2_ (CO_2_RR) represents a promising route to produce carbon‐neutral fuels and chemical compounds.^[1]^ Copper‐based materials are unique at catalyzing CO_2_RR to multi‐carbon hydrocarbons and oxygenated species (C_2+_),^[2]^ while improving the C_2+_ selectivity and proton‐coupled electron transfer kinetics remain key challenges.^[3]^ Importantly, CO_2_RR selectivity and activity depend on the catalyst structure,^[4]^ surface composition,^[5]^ and low‐coordinated surface sites including steps with kink and corner sites^[6]^ or grain boundaries.^[7]^ To enhance C_2+_ selectivity, Cu catalysts have been prepared by engineering low‐coordinated sites and defects, tuning the oxidation state and metal compositions, or modifying their size and shape.[^5^, ^8^] However, to rationally design robust, active and selective CO_2_RR catalysts, nanoscale structure–property relationships derived under reaction conditions are urgently required.^[9]^

Disentangling structural and catalytic properties proves to be intricate due to the dynamic nature of copper electrocatalysts with their structure, morphology, composition, and oxidation state becoming altered during CO_2_RR.^[10]^ Previous works showed the dynamic evolution of Cu nanoparticles^[11]^ and electrode restructuring induced by electrochemical potentials^[12]^ or surface‐adsorbed species.^[13]^ Studies on Cu model surfaces bear the potential of unravelling structure–property relations and the mechanisms behind the observed facet‐dependent product selectivity.^[14]^ In this regard, the square atomic configuration of Cu(100) surfaces was reported to provide optimal adsorption geometry for CO dimers and charged intermediates resulting in enhanced CO_2_RR selectivity toward ethylene,^[15]^ while Cu(111) and Cu(110) preferably produce methane and ethanol, respectively.[^2b^, ^16^] In addition, the beneficial effect of (111) and (110) steps in Cu(100) facets for the carbon−carbon bond formation was reported.^[17]^ Nevertheless, the Cu electrode pretreatment and exposure to ambient conditions or electrolyte was shown to impact the electrocatalytic selectivity and activity.^[18]^ This emphasizes the need for in situ/operando real‐space structural and morphological information of Cu electrodes.^[19]^

While cyclic‐voltammetry‐based studies can provide important information on the catalyst structure, they remain indicative in resolving morphology dynamics or adsorbate‐induced reconstructions. On the other hand, X‐ray techniques provide access to structural information down to minute changes in lattice parameters under operating conditions,^[20]^ but their averaging nature prohibits local characterization. Progress has been made in the understanding of CO_2_RR by combining for instance bulk‐sensitive X‐ray absorption spectroscopy with in situ electrochemical scanning electron microscopy (SEM).^[21]^ Yet, alternative local‐imaging methods must be sought, such as scanning probe microscopy, in order to study catalyst surfaces in situ with local spatial resolution down to the atomic scale. Scanning tunneling microscopy (STM) has provided key insights into the surface chemistry and corrosion of copper.^[22]^ However, while it has been used to follow structural changes of Cu electrodes under applied potential,^[22e]^ STM was carried out mostly in basic and acidic electrolytes and in potential regimes below the onset of CO_2_RR due to experimental limitations.[^22b^, ^22d^] Electrochemical atomic force microscopy (EC‐AFM) has been applied to a much lesser extent to study growth and dissolution processes on (electro)catalyst surfaces^[23]^ and at copper‐electrolyte interfaces in particular.^[24]^ So far, neither EC‐AFM nor EC‐STM have been reported on copper electrodes during CO_2_RR in relevant electrolytes and at highly gas‐evolving potential regimes.

Herein, we provide for the first time in situ nanoscale insight into the intricate structural transformations undergone by a Cu(100) surface during CO_2_RR through EC‐AFM imaging. We address morphological modifications from the as‐prepared state of the copper electrocatalyst through the contact with the electrolyte at various cathodic potentials. Surface line‐defect population and adsorbate‐induced surface structures are characterized from the nanometers scale down to atomic resolution.

## Results and Discussion

**Figure 1:**
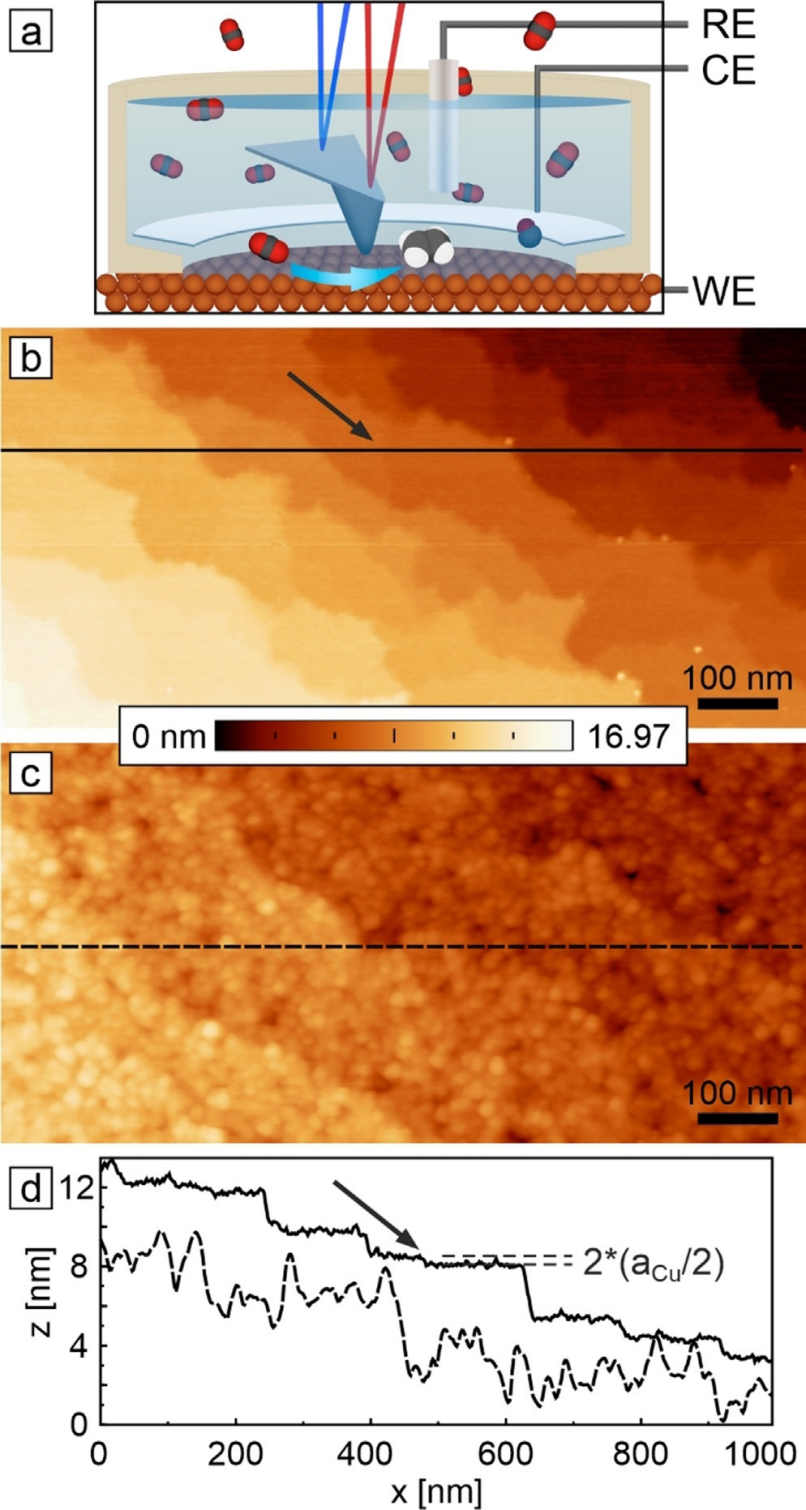
EC‐AFM of hydrothermal oxidation of as‐prepared Cu(100) electrode surfaces. a) Schematics of the in situ AFM cell and the electrochemical setup. In situ AFM images of electropolished Cu(100) recorded in b) air and c) after immersion in CO_2_‐saturated 0.1 m KHCO_3_ at open‐circuit potential (OCV). Line profiles taken at the locations indicated by horizontal lines in (b) and (c) are shown in (d). The arrows in (b,d) mark an atomic double step. Size: 500 nm×1000 nm.

In situ AFM measurements were performed in an electrochemical cell as illustrated in Figure 1 a. In order to conform to standard electrode preparation procedures, Cu(100) single crystals were electrochemically polished.^[25]^ Figure 1 b shows the electropolished Cu(100) surface imaged in air. Such surfaces feature terraces separated by steps or step bunches from atomic scale to several 10 nm height. We selected an image frame with smaller step bunches to also visualize an atomic double step. The measured minimum step height of (184±13) pm agrees with the Cu(100) interlayer spacing (180.5 pm). These step edges represent undercoordinated surface sites associated with crystallographic microfacets. Fine granular structures are observed on the smooth electrode surface after extended exposure to air, suggesting a uniform coverage by a less than 1 nm thin native oxide film (Figure S1a). Equivalent heterogeneous granular structures are observed on copper electrodes prepared in ultra‐high vacuum (UHV) and can be explained by heterogeneous nucleation of oxide phases during the initial Cu(100) oxidation (Figure S1b).^[26]^

**Figure 2:**
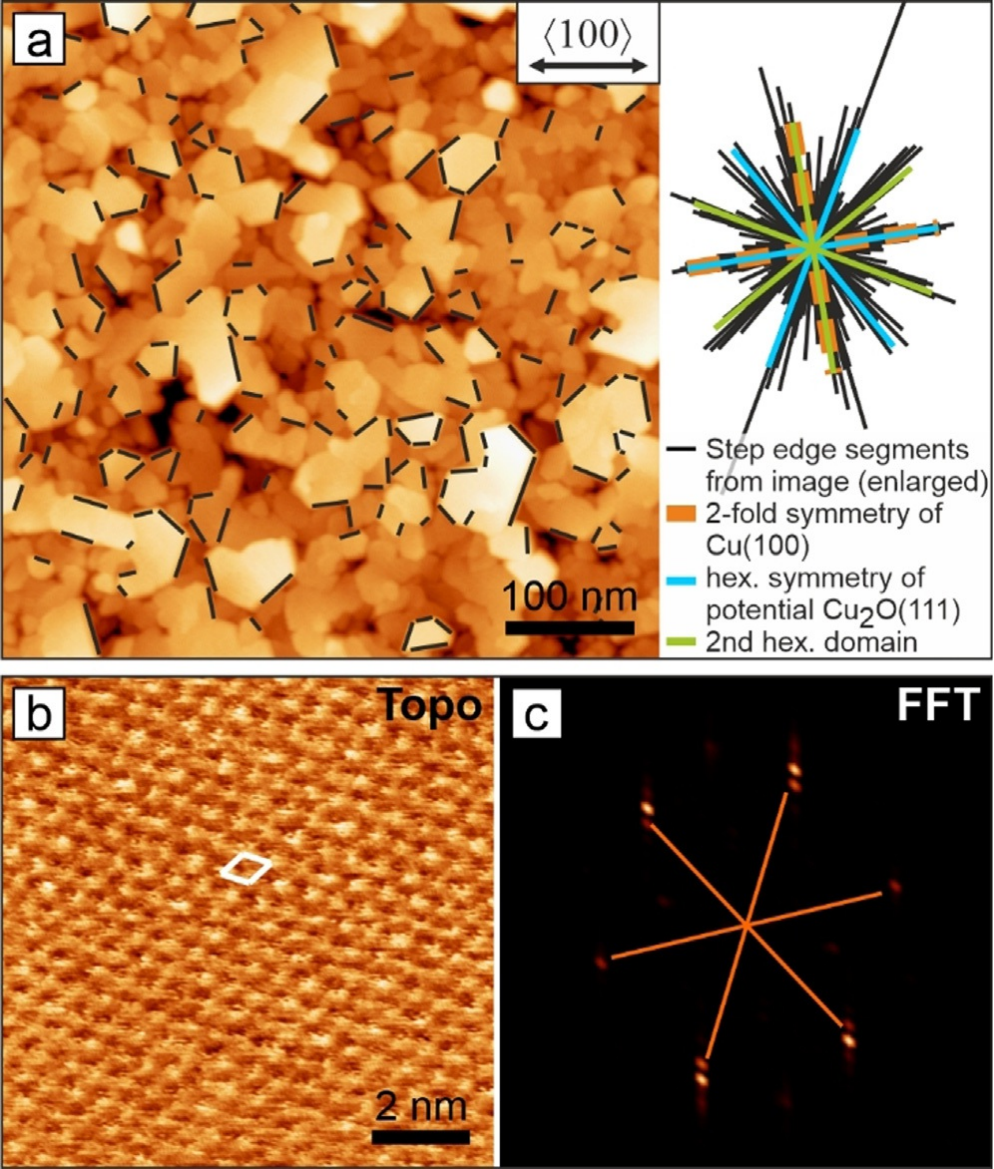
In situ AFM image of Cu_2_O platelets on a Cu(100) electrode in 0.1 m KHCO_3_ at OCV. a) Overview image of the morphology showing epitaxial orientations of the platelets. b) Atomically resolved area on a platelet surface. The white diamond indicates the surface unit cell. c) FFT of (b) showing 0.6 nm hexagonal periodicity.

In the next step, the morphological changes of the copper electrodes were studied upon exposure to a prototypical CO_2_‐saturated 0.1 m KHCO_3_ aqueous electrolyte. Figure 1 c shows the as‐wetted Cu(100) surface at open circuit voltage (OCV).^[27]^ Terraces are evenly covered by much larger globular particles or even bigger platelet‐shaped crystallites (Figure 2 a), while only macro‐steps from the as‐prepared morphology remain visible. Line‐profiles (Figure 1 d) show an increase of height variation on terraces from the as‐prepared (<1 nm) to the as‐wetted surfaces (few nanometers). We identify this native oxide morphology as the duplex film described for wet oxidized copper surfaces.^[22f]^ Due to the solubility of the copper oxide and hydroxide phases, the overlayer is covered by three‐dimensional particles caused by the re‐precipitation of dissolved copper species. Macro‐step bunches down to 4 nm height remain visible after immersion in the electrolyte, which compares well to the thickness of the passive film typically described to be below 10 nm (Figure S2). Similar morphological changes are observed for Cu(100) electrodes exposed to pure water (Figure S3).

While the structure of the granular particles remains to be experimentally resolved, we were able to obtain insight down to the atomic scale on the platelet‐shaped crystallites in 0.1 m KHCO_3_ at OCV, Figure 2. We found the platelet edges to be aligned along twelve distinct azimuthal orientations, which indicates an epitaxial relationship to the Cu(100) substrate organized in two symmetry‐equivalent domains (Figure 2 a). Each domain grows with one hexagonal axis aligned to a closed packed Cu<110> axis. Atomic resolution images (Figure 2 b,c) of the platelet surface show a defect‐free hexagonal pattern with lattice constant *a*=0.6 nm which closely resembles the value obtained for a Cu_2_O(111) bulk‐crystal surface in UHV. Step heights are multiples of the literature value 0.25 nm.^[28]^ Therefore, we assign the platelet morphology to Cu_2_O crystallites. The observation of Cu_2_O(111) structures on top of the native oxide on Cu(100) is noteworthy in the light of previous EC‐STM work in NaOH describing epitaxial CuO(001) to be the topmost native oxide underneath a nanoparticulate overlayer.^[22f]^ It has also been found that interfacial copper(I) oxide grows with Cu_2_O(001)∥Cu(001), while no Cu_2_O(111) surface structures were observed. This altered epitaxial relation could be explained by electrolyte effects and altered surface energetics at different pHs, respectively.

**Figure 3:**
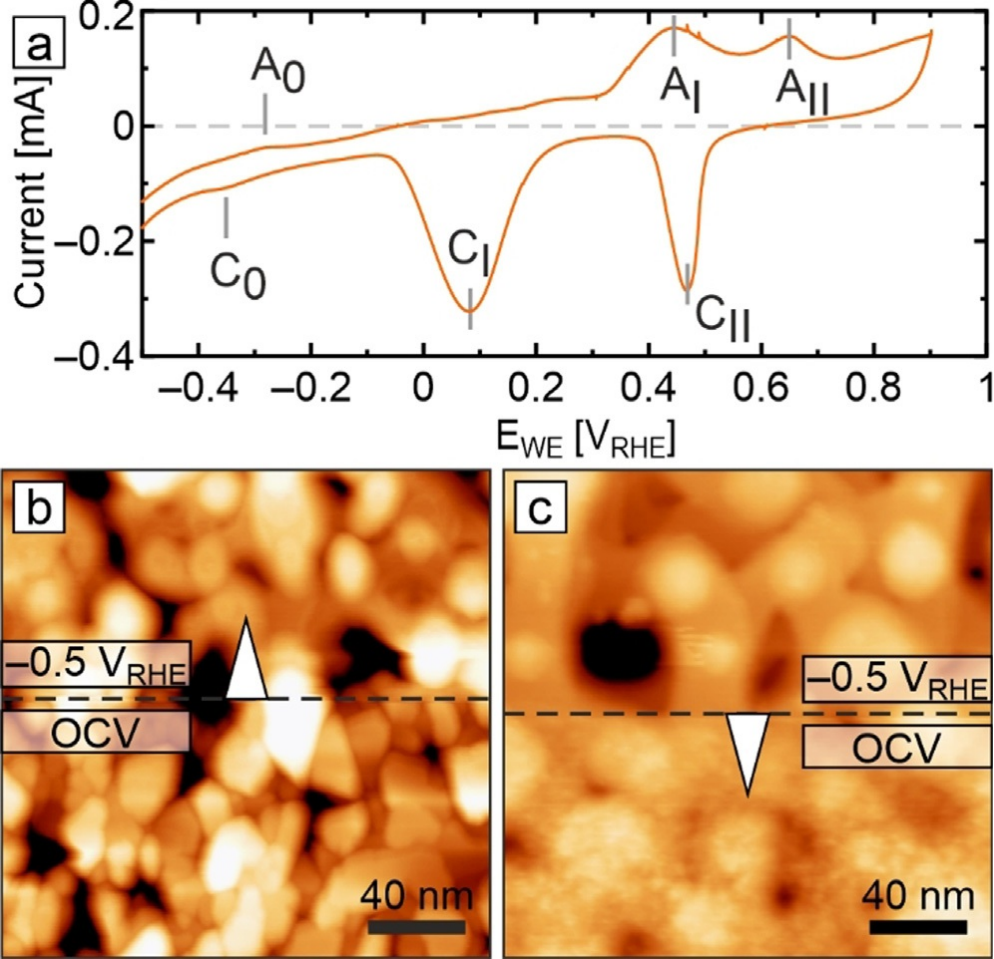
Cyclic voltammogram and in situ AFM images of electropolished Cu(100) in 0.1 m KHCO_3_. a) Cyclic polarization curve recorded in the EC‐AFM with indicated oxidation and reduction features. The full data set is shown in Figure S5. Scan rate: 50 mV s^−1^. In situ EC‐AFM images showing b) the transition from the as‐wetted state (after 20 minutes at OCV) to −0.5 V_RHE_, and c) the reverse transition from −0.5 V_RHE_ to OCV covering the first minute of oxidation.

Taking the as‐prepared and as‐wetted (OCV) surface as starting point, we next focus on the in situ characterization of the Cu(100) catalyst morphology under cathodic polarization. The electrochemical potentials in this work are referenced against the reversible hydrogen electrode (RHE) and range from −0.5 to −1.1 V_RHE_, covering the most relevant CO_2_RR operating regimes for copper‐based catalysts.^[25b]^ Figure 3 a shows a cyclic voltammogram (CV) of Cu(100) recorded in the EC‐AFM cell (for a full‐potential‐range CV see Figure S5). Oxidation peaks A_I_ and A_II_ are associated with the oxide‐formation steps Cu^0^→Cu^I^ and Cu^I^→Cu^II^, while the reduction peaks C_I_ and C_II_ result from the transitions Cu^II^→Cu^I^ and Cu^I^→Cu^0^, respectively. The pair of adsorption/ desorption peaks A_0_ and C_0_ near −0.35 V_RHE_ stems from specifically adsorbed anion species as discussed later. While the CVs are structure‐sensitive, they require side‐by‐side structural information for a conclusive interpretation. In the following, we employ EC‐AFM to address the structural and morphological aspects by imaging catalyst surfaces at specific fixed potentials of interest. Figures 3 b,c shows the transitions of the as‐wetted surface under a potential step between OCV and −0.5 V_RHE_. As revealed in Figure 3 b, reduction of the native oxide film on Cu(100) occurs swiftly and readily. The electrode surface reaches a new steady state within minutes, while large morphological changes occur already within a few scan lines, that is, within several seconds. Figure 3 c highlights that switching off the applied potential results in equally rapid morphological roughening due to oxide overgrowth. Such immediate morphology transformations have to be kept in mind when deriving structure–property relationships based on ex situ and quasi in situ experiments.

**Figure 4:**
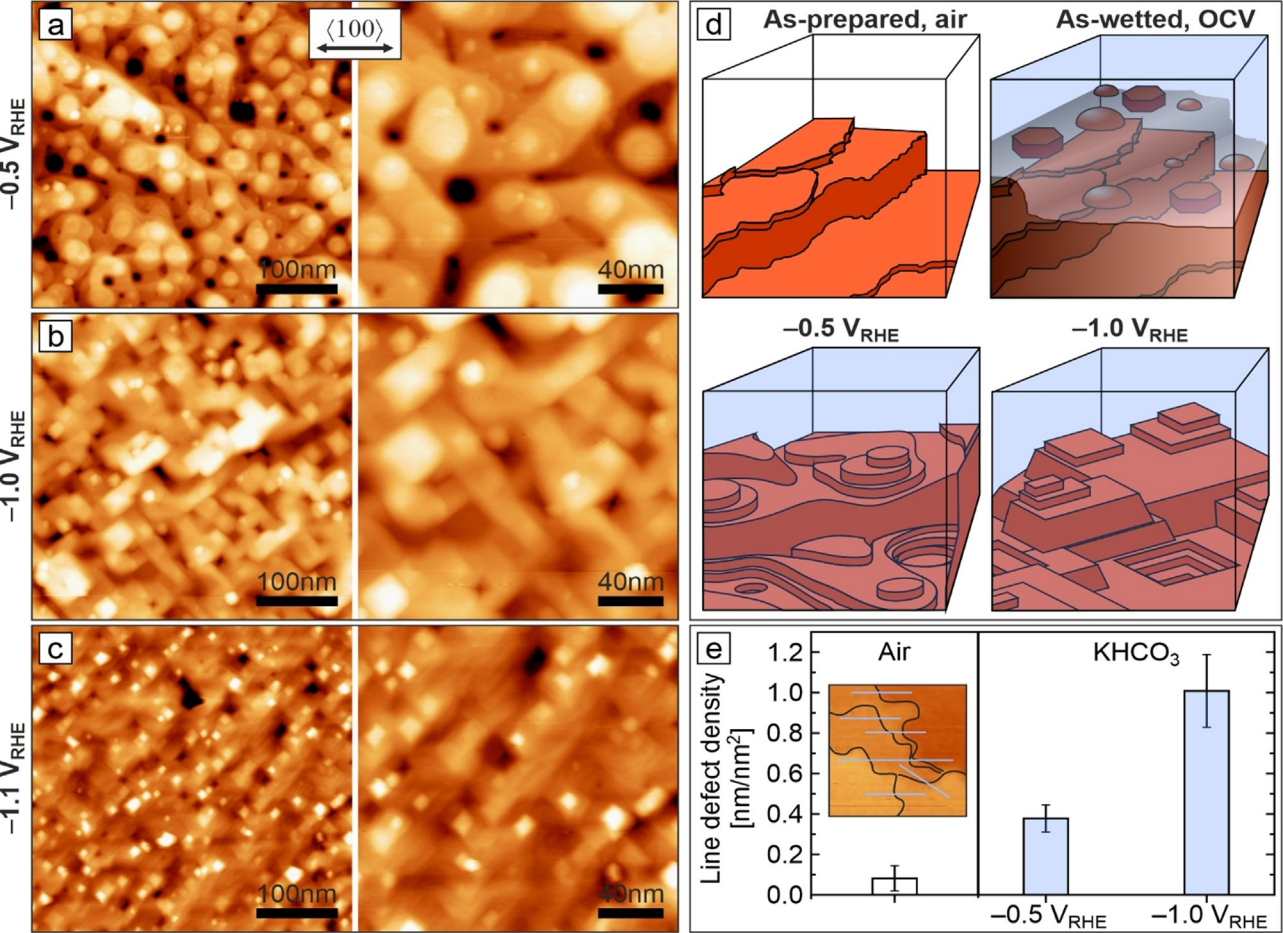
Surface morphology of Cu(100) under different applied potentials during CO_2_RR. a–c) In situ EC‐AFM images of electropolished Cu(100) in CO_2_‐saturated 0.1 m KHCO_3_ recorded at −0.5, −1.0, −1.1 V_RHE_. Image pairs at each potential show an overview and a magnified image. The surface morphologies resulted from cathodic‐step electroreduction of as‐prepared surfaces after immersion at OCV. d) Cross‐section schematics of morphologies observed in this study. e) Bar diagram of the line‐defect densities extracted from in situ AFM images at the respective surface conditions. The inset exemplifies the estimation of line‐defect density based on line profiles and step length.

In situ structural and morphological characterization at length scales below 10 nm is needed for the understanding of the CO_2_RR activities and C_2+_ product selectivities reported for Cu‐based electrocatalysts. This will resolve the currently debated role of crystal faceting, terrace and step‐edge sites, specific adsorption (CO, H), and electrolyte. Figure 4 shows representative in situ AFM morphology images recorded during CO_2_RR at −0.5, −1.0, and −1.1 V_RHE_. At an operating potential of −0.5 V_RHE_, the Cu(100) electrode shows a mound–pit morphology with atomically smooth terraces and atomic scale steps or step bunches (Figure 4 a). The latter are reminiscent of the unreduced morphology. We note that, independent of the starting morphology at OCV (globules, platelets), the structures reduce to the same respective morphologies under reaction conditions. Round, smoothly curved islands and terraces dominate the surface, similar to those observed during copper‐growth experiments in UHV.^[29]^ Essential features of these mound–pit surfaces at −0.5 V_RHE_ are depicted schematically in Figure 4 d. Round islands are thought to result from electrochemical annealing, that is, reduced surface barriers at elevated electrode potentials.^[30]^

Scanning probe measurements have been considered to be limited under electrochemical reaction conditions by Faradaic currents and gas evolution interfering with tunneling currents.[^22b^, ^22d^] As a consequence, certain catalytic reactions such as CO_2_RR have been studied only within a limited potential range and selected chemical environments. While gas evolution does not affect our in situ AFM measurements at potentials down to −0.5 V_RHE_, we further explored the electrocatalysts under harsher conditions at more negative cathodic potentials, which are the most relevant for the formation of C_2+_ products such as ethylene.

In contrast to −0.5 V_RHE_, CO_2_ electroreduction at −1.0 V_RHE_ produces a morphology featuring straight terrace edges with right angles, Figure 4 b. Despite the gas evolution, stable in situ imaging down to the atomic scale proves to be possible. Step edges are crystallographically aligned with Cu<110> and now adopt the configuration of equally oriented square islands and pyramid shapes that replace the round mounds and pits. The number of step bunches appears to increase as compared to the smoothly curved shape at −0.5 V_RHE_, pointing towards a raised Ehrlich–Schwoebel barrier. Altered step‐edge dynamics based on different kink‐formation energies explain the straight terrace edges.^[29]^ The round and rectangular island and mound shapes in turn indicate qualitative changes in the nature of the step sites. Curved structures will contain more kink sites than straight edges. Step edges constitute low‐coordination sites with altered chemical properties, depending on their geometric atomic structure (type of microfacet, kink or corner site). They are considered to play a central role in structure–activity/selectivity relations.[^5^, ^7^, ^13e^, ^31^] Theory has found favorable step structures for CO_2_RR on copper.[^17^, ^32^] Hence, the observed morphology changes are expected to directly impact CO_2_RR selectivity.

Reduction at −1.1 V_RHE_ produces a morphology similar to that at −1.0 V_RHE_ with the surface exposing Cu(100) terraces. However, the observed rectangular structures appear smaller in size, which might already be an expression of cathodic corrosion.^[33]^ At this negative potential, gas evolution has noticeable effects on AFM imaging, requiring an increased scan speed (Figure S8). At all three potentials, the observable monoatomic step heights of (188±21) pm in the electrolyte are comparable with the 180.5 pm interlayer spacing of Cu along <001>. This is in line with the assumption that the surface is mainly composed of metallic copper under such reducing conditions.^[21]^ Direct insight into effects of CO_2_ and CO_2_RR products onto surface morphology can be found in data recorded in CO_2_‐free Ar‐saturated electrolyte (Figure S7). Images evidence a reduced electrochemical annealing effect at −0.5 V_RHE_. At identical potential this implies a different degree of specific adsorption, which would be related to CO_2_ and CO_2_RR intermediates. Further operando spectroscopic studies are needed to clarify this.

In addition, we performed CO_2_RR at the three selected potentials over extended periods of time while simultaneously imaging the respective morphologies. Figure S4 shows in situ AFM images of as‐wetted Cu(100) across cathodic potential steps to −0.5, −1.0, and −1.1 V_RHE_, as well as snap‐shots after 30 minutes and 90 minutes of CO_2_RR. Such images reveal no obvious morphological changes on the surface over the reaction times studied.

The discussion of defects often remained basic and speculative, introducing unspecified defects to explain changes in the reaction rate and product selectivity. Nevertheless, first attempts have been made to identify the type of defects and the nature of their action in CO_2_RR with electron microscopy on grain boundaries in nanoparticles and infrared spectroscopy (ATR‐FTIR) of CO on undercoordinated sites.[^7b^, ^13e^, ^34^] The most simple and prominent surface defects present in any real surface are straight terrace edges modified by kinks and corner sites. Due to their abundance, they may be more relevant than, for example, grain boundaries or dislocations, which are limited by the diffusion to the defect sites through point‐ and line‐shaped openings in the surface. However, in order to disentangle the various defect types experimentally and to assess their presence and effect, the catalyst surfaces must be characterized in real space under reaction conditions. In situ AFM can provide high‐resolution information on the nature of the defects and prevalence under a given electrochemical potential, with advantages in identification and surface sensitivity over ATR‐FTIR and electron microscopy.^[21]^

To this end, we compared step‐edge density estimates for copper surfaces during CO_2_RR at different reducing potentials with those for as‐prepared samples and found variations in line‐defect densities as a function of the sample state (Figure 4 e). As‐prepared electropolished Cu(100) electrodes exhibit the lowest defect densities. Changes induced by oxidation and dissolution–precipitation in the as‐wetted state roughen the catalyst surface visibly (compare Figures 1 and 4 a–c), which is reflected in elevated line‐defect density values for the reduced surfaces, Figure 4 e. Of special interest is the qualitative change in surface morphology, round at −0.5 V_RHE_ versus rectangular at −1.0 and −1.1 V_RHE_, accompanied by another increase in line‐defect density. We tentatively explain this with the smoothing effect of the electrochemical “surface healing” at less negative potentials. Our data reveal the preservation of some of the three‐dimensional morphology from the as‐wetted state and provide potential CO_2_RR active sites for adsorption, dissociation and C−C coupling on the terrace edge or neighboring microfacets. Step‐edge orientation and faceting are closely linked to surface adsorption and pose an important source of information on the structure of low‐coordinated sites.^[35]^ In contrast to deaerated electrolyte, non‐deaerated electrolyte produces quadratic islands instead of smoothly curved step edges at −0.5 V_RHE_ (Figure S6). Terrace edges are aligned along the energetically less stable Cu<100> axis, but change their orientation to become aligned along the closed‐packed Cu<110> axis when the potential is set to −1.1 V_RHE_. This is indicative of the presence of adsorbates which are absent at more negative potential. Adsorbates with unit cell size and symmetry different from that of the metal surface can stabilize step edges along deviating crystal axes.^[35]^ Edges follow Cu<110> on bare copper, but align along Cu<100> for the (2√2×√2)R45°‐O surface oxide and for c(2×2) halide or nitrogen ad‐structures, being their close‐packed axes. Thus, the observed 45° rotation for the straight step edges in deliberately aerated electrolyte may be explained by adsorbed oxygen.

Work on Cu_2_O‐derived and low Miller index single‐crystal Cu electrodes revealed a systematic offset between CO evolution and C_2+_ production of 300–400 mV to more negative potential and a clear suppression of methane formation on the presumably rougher Cu_2_O‐derived Cu electrodes.^[16f]^ This suggests kinetic effects of the CO adsorbate to be the rate limiting step, which leads to questions regarding the optimum adsorbate coverage, interference of co‐adsorbates, CO–CO coupling and the stable morphological structures at different applied potentials under operating conditions.[^16c^, ^17^] Spectroscopic observation of CO‐induced surface restructuring or reconstruction at elevated CO concentrations further underlines the need for real space information.^[13e]^

**Figure 5:**
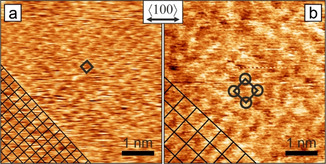
Atomic resolution in situ AFM images of electropolished Cu(100) surfaces under different reducing potentials in CO_2_‐saturated 0.1 m KHCO_3_. a) At −1.0 V_RHE_ a (1×1) surface is present. b) At −0.5 V_RHE_ a p(2×2) superstructure has been observed. Unit cells are indicated by black squares. The double arrow marks the nominal bulk copper <100> direction for both frames, actual alignment is indicated by the square lattices in the bottom left corners. Both images: 5 nm×5 nm, unfiltered data.

To better understand morphology and surface termination, we obtained atomically resolved in situ AFM images on Cu(100) surfaces. Figure 5 shows the resolved surface terminations and clearly reveals that different structures may appear at distinct cathodic potentials. The periodicity of the primitive square lattice in Figure 5 a imaged at −1.0 V_RHE_ is 260 pm, matches that of bare copper (255.6 pm), and represents an (1×1) surface termination. The common step height of (188±21) pm further underpins the presence of a bare surface or at most a thin homogeneous surface adsorbate. Candidates for adsorbed anions on Cu(100) in aqueous KHCO_3_ are oxygen, hydroxyls, carbonate,[^13e^, ^36^] formate,^[37]^ hydrogen,^[38]^ carbon monoxide,^[39]^ a H+CO coadsorbate,^[11c]^ as well as metal impurities in the electrolyte and sulfur from the bulk of copper. The key adsorbate and essential intermediate on copper under CO_2_RR conditions in KHCO_3_ is CO. Depending on its concentration, different coverages and effects on the morphology have been observed.[^13d^, ^39c^] However, the reported CO coverages for CO_2_RR at −1.0 V_RHE_ are incompatible with dense (1×1) ad‐layers. This does not exclude presence of CO at low coverage as tip‐induced lateral motion may prevent imaging until a complete monolayer is reached. Very high CO concentrations could also induce roughening, which is obviously not the case.^[13e]^

The structure in Figure 5 b recorded at −0.5 V_RHE_ has twice the lattice period of copper, which is compatible in size and orientation with a p(2×2) superstructure. This is a clear indicator of specific anion adsorption, as no reconstruction of bare Cu(100) is known in this potential regime or in UHV. Even though the wide superstructure lattice prohibits ion‐size‐based exclusion of any of the ad‐species mentioned earlier, primitive p(2×2) structures have been reported only for CO in UHV,^[40]^ sulfur in UHV and electrolyte,^[41]^ as well as second‐layer metal surface alloys.^[42]^ The p(2×2) pattern at −0.5 V_RHE_ can be understood as a low coverage CO phase preferred over the more dense c(2×2) structure, in line with CO concentrations from ATR‐FTIR. The fact that the p(2×2) surface at −0.5 V_RHE_ in Figure 5 b is not always observed may again be due to a sub‐monolayer coverage. A p(2×2) is also compatible with hydrogen co‐adsorption at more negative potential, although this has not been observed so far. While CO is the focus of interest, contaminants or other low‐coverage intermediates cannot be ignored. Sulfur could be the origin of the small adsorption/desorption feature at −0.3 and −0.4 V_RHE_ in the CV, as reported for an acidic electrolyte, and may produce a p(2×2) reconstruction.^[43]^ This finding is of relevance for samples prepared outside UHV, without depleting the near‐surface region from common impurities, which is the case for electropolished samples. We tentatively conclude that CO intermediates may be imaged in the CO_2_RR regime if complete monolayers form. Nevertheless, further chemical information is needed to ascertain this. The presented atomic surface structures have intimate links to the ongoing reaction, impurities, potential, step‐edge orientation, and morphology. Unraveling these interdependencies in CO_2_RR on copper requires morphological and structural information together with spectroscopic data to correctly interpret activity and selectivity.

## Conclusion

Herein, we have unraveled in situ and in real space the morphology changes and surface reconstruction of a model Cu(100) electrocatalyst during the electrochemical CO_2_ reduction. Operating electrochemical AFM in highly gas‐evolving reaction environments enabled the direct observation of oxidized copper surfaces entering different morphological regimes when certain cathodic CO_2_RR potential ranges are set. Particularly, we have observed at the atomic scale (i) an epitaxial Cu_2_O(111)/Cu(100) phase formed at open circuit potential; (ii) morphological transformation occurring on a second time scale to smoothly curved (−0.5 V_RHE_) and rectangular terraced catalyst surfaces (−1 V_RHE_), respectively; paired with (iii) structural transitions to specific adsorption‐induced p(2×2) reconstructions and (1×1) Cu surfaces; and (iv) increasing density of undercoordinated Cu sites (step edges) with higher cathodic starting potentials.

These results directly reveal the intricate interplay between selectivity‐determining factors in copper‐based CO_2_RR: catalyst preparation and handling, morphology/ structure, defect nature and density, applied potential, and electrolyte. While previously metallic (1×1) Cu surfaces have often been assumed under operating conditions, this work specifically shows that surface reconstructions over relevant cathodic potentials need to be considered in order to establish valuable structure–property relationships in CO_2_RR. Our study indicates potential‐dependent qualitative changes in the nature and density of Cu step sites with implications on the CO_2_RR selectivity of Cu‐ and Cu‐oxide‐derived single‐crystalline, polycrystalline and nanoparticulate CO_2_RR electrocatalysts. The use of state‐of‐the‐art in situ EC‐AFM gives new impetus to the in situ characterization of a broader class of catalytic materials under relevant reaction conditions, including non‐conducting ones, and stimulates comprehensive theoretical investigations on adsorbate‐ and potential‐induced restructuring, as well as synthetic strategies toward achieving improved catalytic performance and stability.

## Conflict of interest

The authors declare no conflict of interest.

## Supporting information

As a service to our authors and readers, this journal provides supporting information supplied by the authors. Such materials are peer reviewed and may be re‐organized for online delivery, but are not copy‐edited or typeset. Technical support issues arising from supporting information (other than missing files) should be addressed to the authors.

Supplementary
