# Supplementary material for: Potential‐Dependent Morphology of Copper Catalysts During CO2 Electroreduction Revealed by In Situ Atomic Force Microscopy
Source: Angew Chem Int Ed Engl. 2020 Dec 1;60(5):2561–8. doi: 10.1002/anie.202010449 (PMC7898873; doi:10.1002/anie.202010449)
Supplement: Supplementary file 1 — Supplementary [file ANIE-60-2561-s001.pdf]

Angewandte Chemie
Eine Zeitschrift der Gesellschaft Deutscher Chemiker

Supporting Information

# Potential-Dependent Morphology of Copper Catalysts During $CO_2$ Electroreduction Revealed by In Situ Atomic Force Microscopy

*Georg H. Simon, Christopher S. Kley,* and Beatriz Roldan Cuenya*

anie_202010449_sm_miscellaneous_information.pdf

## Supporting Information

**Content:**


1. Experimental methods
2. As-prepared: Comparison of electropolished and UHV-prepared Cu(100) after air exposure
3. Height scale of morphological changes
4. Electropolished Cu(100) in deaerated $H_2O$
5. Morphology of Cu(100) under static potential over time
6. Cyclic voltammogram of electropolished Cu(100) in 0.1 M $KHCO_3$
7. Effect of specific adsorption on surface morphology
8. Cu(100) in argon saturated 0.1 M $KHCO_3$
9. Adsorbates on Cu(100)
10. Gas evolution in the EC-AFM cell during imaging
11. Supplementary references

## 1. Experimental methods

***Preparation of single crystals:*** Single crystalline, mirror polished, high purity (6N) Cu(100) samples for UHV studies were purchased (MaTecK GmbH). Samples surfaces were oriented to within 0.1-0.3° to Cu(100) and had one in-plane <100> axis marked for oriented mounting in the EC-AFM. After UHV preparation by argon ion sputtering and annealing, the crystals were imaged in the EC-AFM in air, and exhibited flat and extended terraces separated by step edges ranging from monoatomic to a few atom layers height.

Electropolished samples (EP) were prepared after the initial UHV experiments by electropolishing the aforementioned crystals in pure 85% ortho-phosphoric acid, $H_3PO_4$ (PanReac, AppliChem), or a mixture of $H_3PO_4$+$H_2SO_4$+$H_2O$ (98% $H_2SO_4$: EMSURE, Sigma-Aldrich) at 3 V for up to 3 minutes followed by washing in ultrapure $H_2O$ and drying in nitrogen gas (5N) flow. A basket type sample holder and cylindrical counter electrode made of copper wire and foil respectively were used for electropolishing in a two electrode configuration. No differences in sample surface were observed between the two polishing baths. The samples were transferred to the EC-AFM through room air.

***EC-AFM:*** AFM and EC-AFM images, in air and *in situ* in electrolyte/water, were obtained in amplitude modulation (AM-AFM) mode also known as intermittent contact mode. The commercial AFM (CypherES, Asylum Research / Oxford Instruments) is a sample scanning system equipped with photo-thermal excitation of the cantilever oscillation [1] and optical detection (405 nm, 850 nm) as well as a closed sample compartment to control the atmosphere surrounding sample and electrolyte. High frequency cantilevers (ARROW UHF Au, NanoWorld AG) were used throughout this study after cleaning with a gentle argon plasma treatment prior to imaging. Spring constants of the used cantilevers were (8±5) N/m and resonance frequencies were in the range of (1200±300) kHz in air and (440±110) kHz in water or electrolyte. The used oscillation amplitude set points fall in the range of 0.25-7 nm in air and 0.1-5 nm in water and electrolyte. Images were tilt corrected only.

***EC-cell:*** Samples can be mounted in an open three electrode electrochemical cell (PEEK, FFKM) within the sealed AFM compartment. Ring shaped platinum sheet (99.996% purity, Asylum Research) and silver wires (99.99% purity, Asylum Research) were used as counter (CE) and reference electrodes (RE) while the sample constitutes the working electrode (WE). The exposed geometric WE surface area amounts to 0.55 $cm^2$, the CE surface area is about twice as large. Electrolyte volumes ranged between 0.2 ml and 0.35 ml.

***Reference electrode:*** Electrochemical potentials were recorded with bare silver wires as reference electrodes and calibrated against RHE (miniHydroflex, Gaskatel) in deaerated and $CO_2$ saturated 0.1 M $KHCO_3$. Calibration was carried out in the open EC-AFM cell without the immersed AFM sensor.

***Potentiostat:*** For chronoamperometric and potentiodynamic measurements a SP-200 potentiostat (BioLogic) equipped with an ultra-low current module was used.

***Atmosphere control:*** Headspace filling was performed in order to counteract de-carbonation of the $CO_2$-saturated electrolyte and to prevent re-oxidation and contamination with nitrogen, sulfur and hydrocarbons. For this purpose $CO_2$ from a stainless steel ($CO_2$: 5.3N) or aluminum ($CO_2$: 4.5N) pressure cylinder is fed through PTFE tubing into the sealed sample compartment of the CypherES at preset overpressure. A moisturizing bottle filled with pure $H_2O$ is added to the line upstream from the sample compartment to prevent electrolyte evaporation.

***Electrolyte:*** Batches of 0.1 molar potassium bicarbonate aqueous solutions were made from high purity $K_2CO_3$ (99.997% metals basis, Puratronic, Alfa Aesar) and ultrapure water (18 MOhm, HiPerSolv Chromanorm, VWR), here denoted as 0.1 M $KHCO_3$. Electrolytes were deaerated and $CO_2$ saturated by feeding high purity argon (5N) and carbon dioxide (4.5N) gas through a PTFE tube to the base of the vessel at noticeable overpressures.

***Defect characterization:*** Step edge densities of as-prepared and as-reduced electropolished Cu(100) samples are given per unit area and were obtained manually as products of step segment length and number of monoatomic steps in a step(bunch) taken from line profiles in tilt corrected images. This implies that step bunches are well represented by step cascades of monolayer-high single steps and not by atomically smooth facets of low Miller index crystal planes.

## 2. As-prepared: Comparison of electropolished and UHV-prepared Cu(100) after air exposure

Electropolished samples show increased mesoscopic roughness and thus an increased step density with smaller terraces. Nevertheless, large flat surface areas can be found on these samples, Figure S1a. Step heights are multiples of the Cu(100) interlayer spacing of 180.5 pm as indicated by dotted lines in Figure S1b. The terraces show a similar structure as in the ultrahigh vacuum (UHV) case (Figures S1c,d). Granular structures cover the entire surface area evenly and in favorable cases are even smaller than those observed on some UHV-prepared samples, as shown in Figure S1. However, due to the altered surface morphology, the electropolished surface shows typically larger step bunches and only a few monoatomic steps.

For comparison, Figures S1c,d shows a UHV-prepared sample with flat surface morphology and evenly distributed terraces of several 100 nm width and step heights down to monoatomic steps (180.5 pm). Even exposure to air moisture for extended periods of time (hours-days) preserves this morphology. The grain sizes observed depend slightly on time and on AFM tip radius (tip convolution effect).

In both cases, the surface passivates immediately and comprehensively upon preparation. The surface morphology of the metallic copper underneath is preserved to a large extend. The resulting terrace roughness is below 0.5 nm. The granular passivation features can be understood from the heterogeneous nucleation of planar, linear and three dimensional oxide phases on Cu(100) at 100 mbar oxygen pressure.[2]

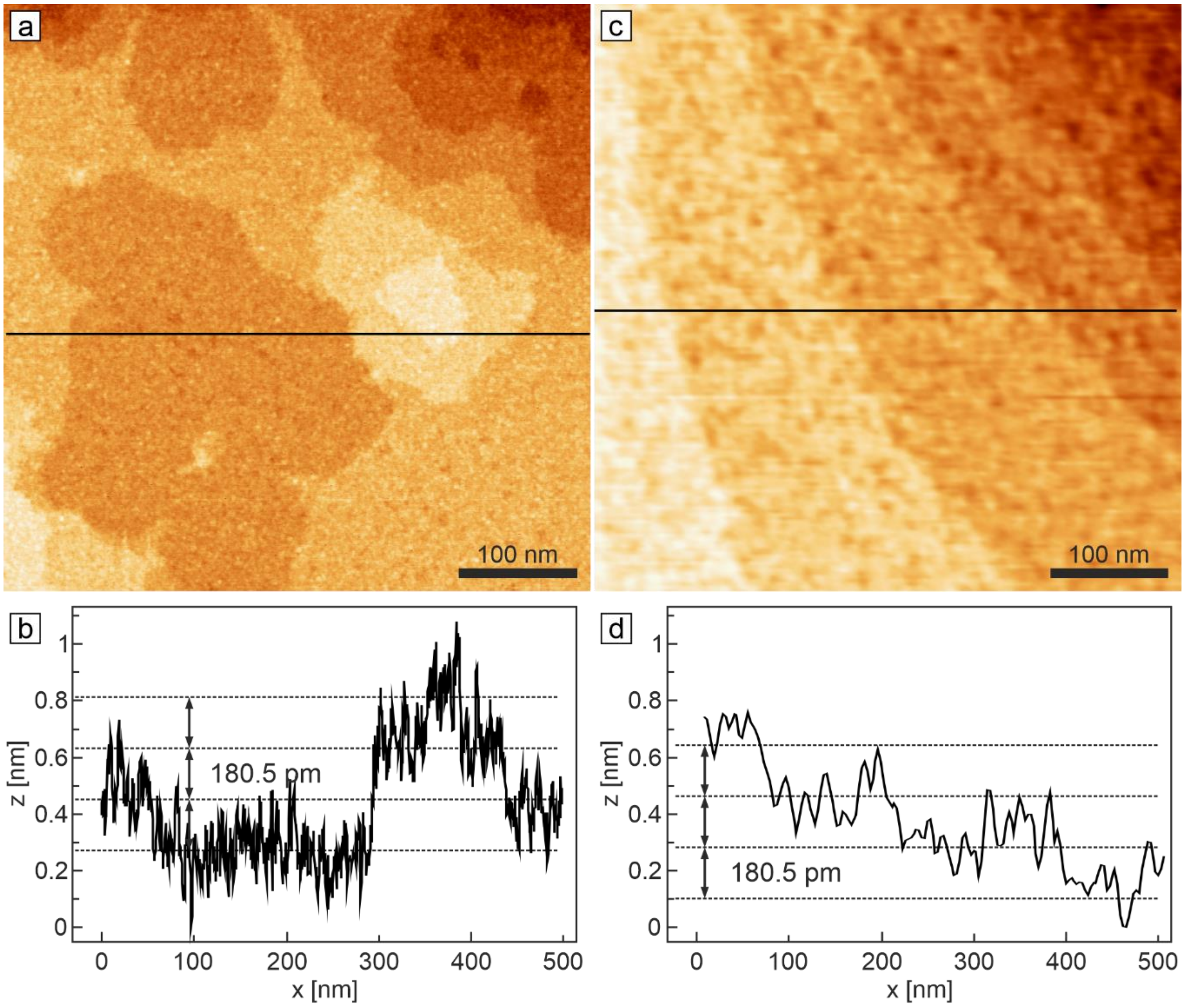


**Figure S1.** Comparison of as-prepared electropolished and UHV-prepared surface terraces in air. (a) AFM image of an electropolished surface after 1.5 h in air. Mono-atomic steps are observed besides step bunches several times that height. Granular structures cover all terraces. (b) AFM image of a UHV-prepared Cu(100) surface after air exposure. Terraces separated by steps of atomic height dominate the surface. Again the entire surface is covered by granular structures rather evenly. The typical terrace roughness is below 0.5 nm in both cases. Equidistant dotted lines represent the Cu(100) interlayer spacing of 180.5 pm. Both images 500 nm × 500 nm.

## 3. Height scale of morphological changes

While AFM topographies do not generally provide a direct measure to gauge the depth to which wet oxidation affects the surface, the comparison of certain morphological features can give some understanding. Figure S2 shows the (a,b) as-prepared and (c,d) as-reduced state of a sample, which had been prepared in UHV after suffering some roughening during electropolishing. The latter resulted in cascades of massive step bunches, up to several 10 nm in height, with accurately aligned step edges. After growth and reduction of the usual hydrothermal oxide layer in the electrolyte. Only the large step bunches are reminiscent of the initial morphology, while the terraces comprise several nanometer or even 10 nm deep pits. This leads us to the conclusion that the observed height variations due to wet oxidation is 15-20 nm. This is not too different from the observed thicknesses of 6-10 nm for passive films grown on copper in other electrolytes.

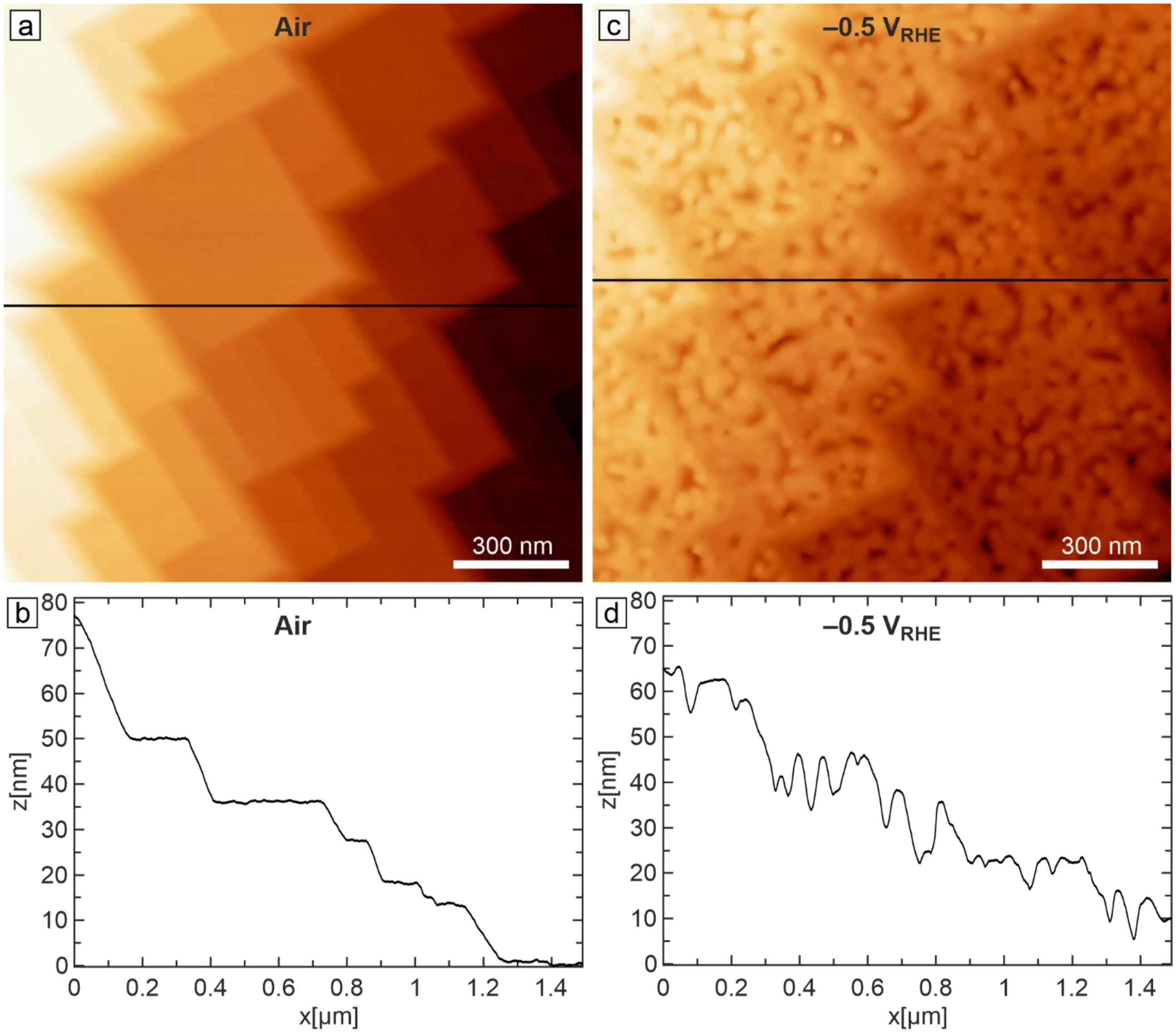


**Figure S2.** Effect of the contact with the electrolyte and subsequent reduction on an electropolished and subsequently UHV-prepared Cu(100) surface. (a) As-prepared surface. Straight step edges aligned with the closed packed <110> axes of fcc-Cu dominate the morphology. The steps are bunches of several nanometers to tens of nanometers in height. See line profile underneath. Granular surface coverage is not visible at this large height variation, but still present. (b) Same sample after contact with the 0.1 M $KHCO_3$ electrolyte at OCV and subsequent reduction at –0.5 $V_{RHE}$. Straight step edges along Cu <110> are reminiscent of the larger step bunches in the initial preparation. Image sizes 1500 nm × 1500 nm.

## 4. *In situ* AFM on electropolished Cu(100) in deaerated $H_2O$

Submerging the electropolished or UHV-prepared copper surfaces within the aqueous electrolyte results in the drastic morphology changes described in the main text. Exposure to pure $H_2O$ produces a similar effect. The *in situ* AFM images in Figure S3 show an electropolished Cu(100) surface in contact with pure water. The granular structures observed and loss of morphological details, like single atomic steps from the as-prepared state, are strikingly reminiscent of the corresponding images in an aqueous bicarbonate solution. Surface roughness is consequently increased to several nanometers also in the case of pure water. Although our electrolytes were composed of highest purity ingredients and deaerated by bubbling with 5N argon for several tens of minutes to many hours (and subsequently with $CO_2$, if not indicated otherwise), we cannot fully exclude residual oxygen which could oxidize the sample surface without an applied electrochemical potential.

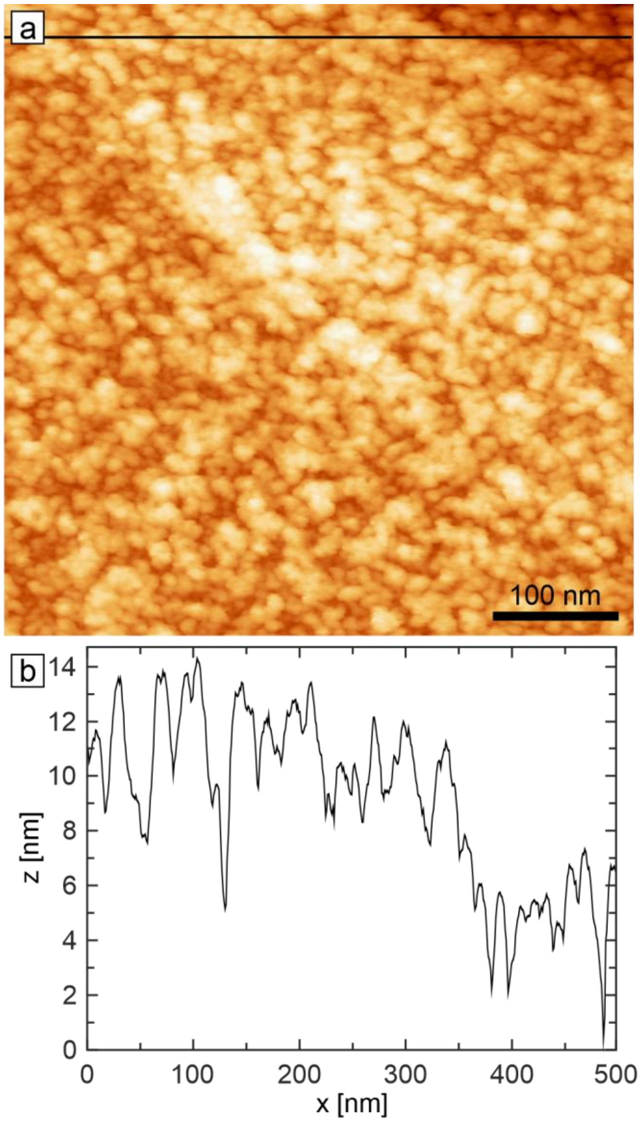


**Figure S3.** *In situ* AFM image of a Cu(100) single crystal electrode recorded in pure $H_2O$. Image size 500 nm × 500 nm.

## 5. Morphology of Cu(100) under static potential over time

As many electrocatalytic experiments evolve over time scales much longer than a series of EC-AFM images, i.e. sometimes many hours or days rather than half an hour, we show a time series in Figure S4 of the morphological development from the initial reducing cathodic potential step to 1.5 h afterwards while being held continuously at the same reducing potential. These *in situ* images witness a rapid change from the nanoparticulate as-wetted surface morphology to the reduced states at three different potentials. Arrows in the images indicate the direction of the AFM and EC scans. The location of their base coincides with the application of the cathodic potential step. Transformations from the as-wetted to the reduced state occur during only a few scan lines, i.e. within several seconds, but completion may take several images, i.e. 5-10 minutes, presumably depending on the thickness of the initial passivating layer. However, the latter impression could also result from poor tip states, which may prohibit good resolution until the interaction with (mobile) surface species might alter again the tip and result in better resolution. After the swift initial transition, the surface reaches a steady state with characteristic morphological features for a given potential. This steady state morphology is stable at least over the course of 1.5 hours.

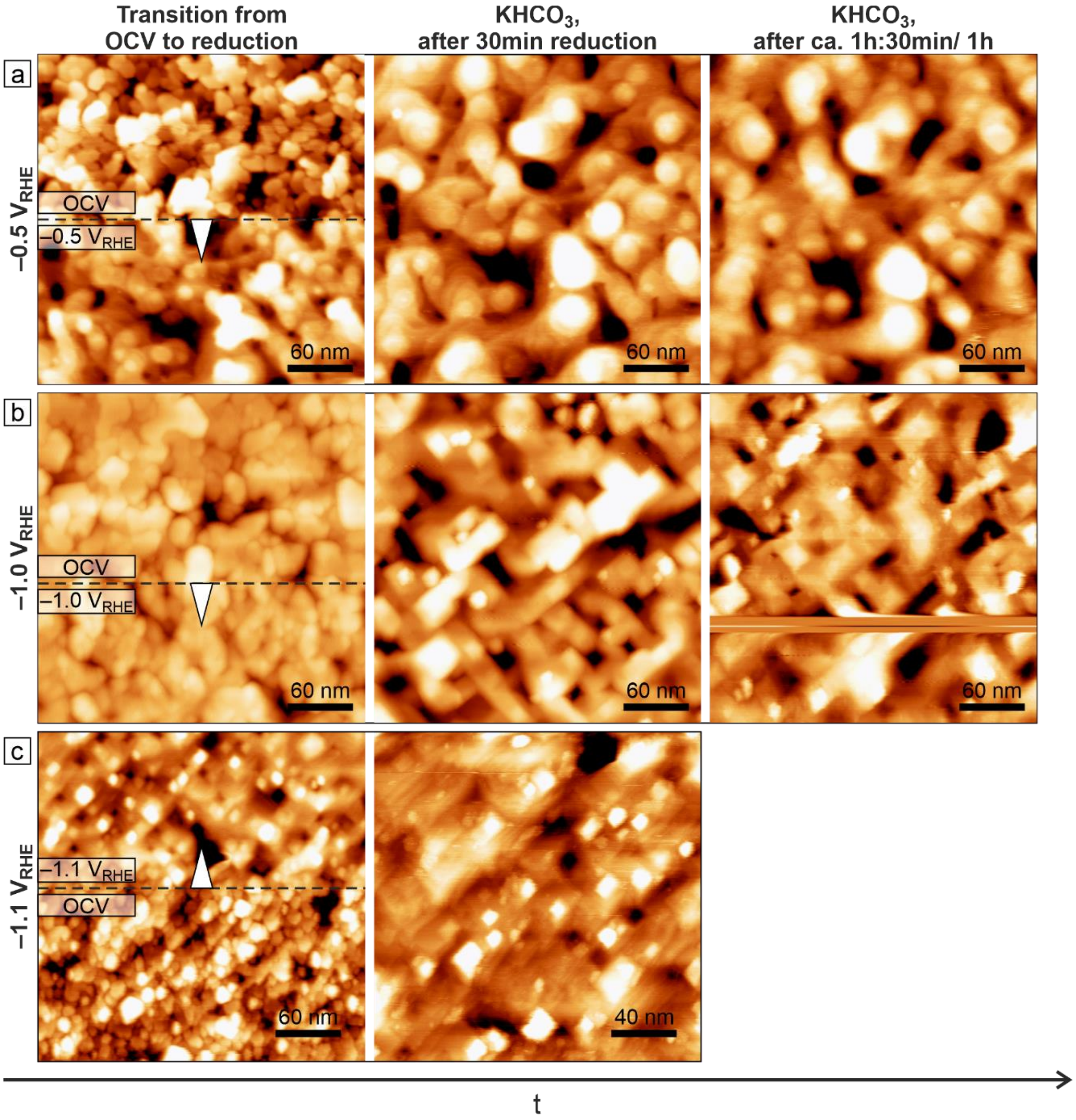


**Figure S4.** *In situ* EC-AFM time series of Cu(100) in $CO_2$-saturated 0.1 M $KHCO_3$ recorded at: (a) –0.5 $V_{RHE}$, (b) –1.0 $V_{RHE}$, and (c) –1.1 $V_{RHE}$. The featureless horizontal stripe in the middle image of the right most column is owed to a bubble temporarily blocking the detection laser. This image has been recorded on a different surface area of the same sample shown in the other images of (b).

## 6. Cyclic voltammogram of Cu(100) in 0.1 M $KHCO_3$

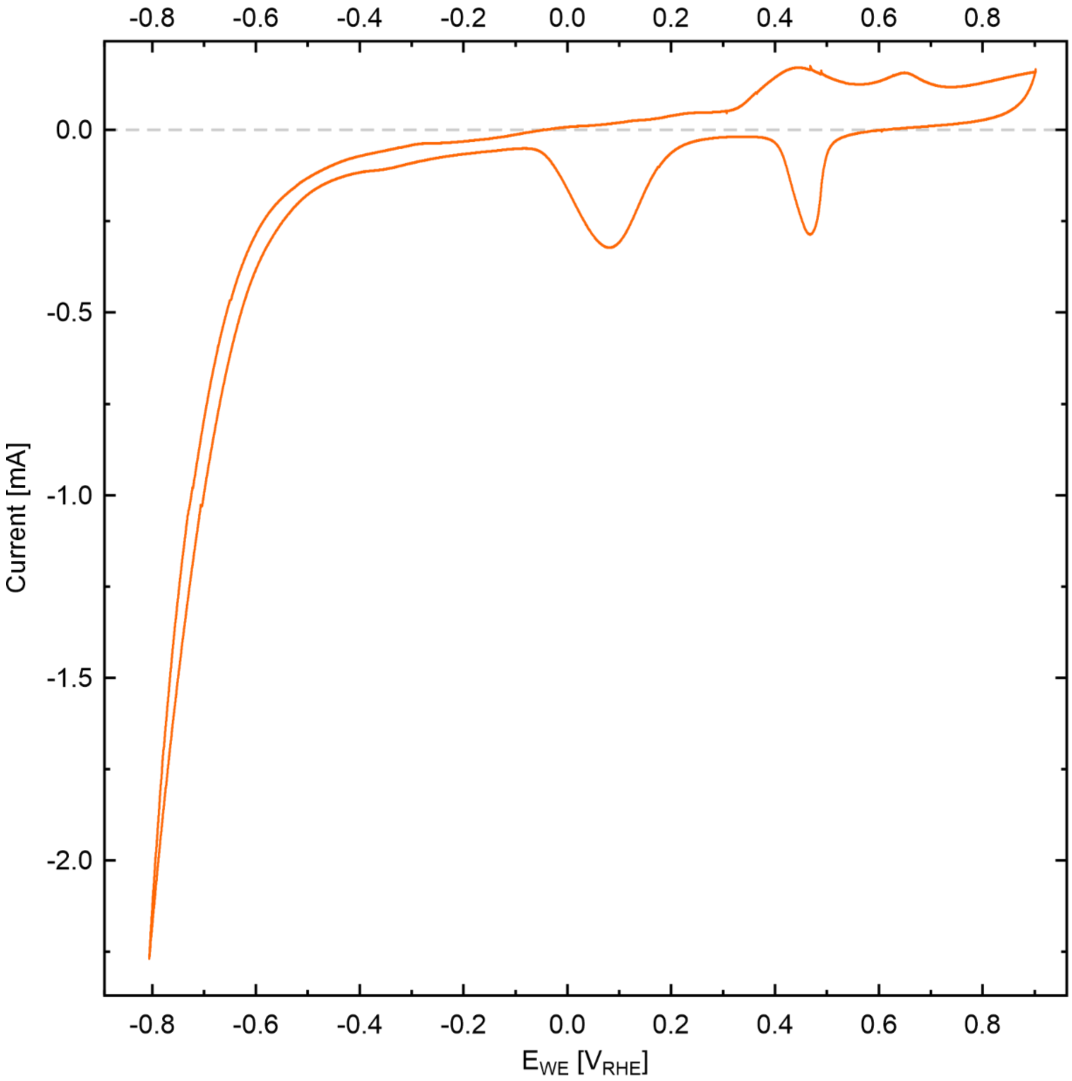


**Figure S5.** Cyclic voltammogram of electropolished Cu(100) in deaerated and $CO_2$-saturated 0.1 M $KHCO_3$ aqueous electrolyte recorded in the EC-AFM at a scan rate of 50 mV/s.

## 7. Effect of specific adsorption on surface morphology

As described in the main text, there are specifically adsorbed anions known to affect the surface structure and morphology. By virtue of strong chemisorption bonds, ordered surface adsorbates can stabilize certain substrate geometries as shown for copper in early EC-STM work.[3] When atomic resolution is not achieved, this knowledge may enable deduction of the surface adsorption state from the surface morphology as illustrated in Figure S6. For non-deaeraed electrolyte and –0.5 $V_{RHE}$ for example, islands and terrace structures with step edges aligned along Cu<100> axes are observed, while deaerated electrolytes produce smoothly curved edges at the same cathodic potential (Figure S6a+b). This is rationalized by pinning of the copper substrate by adsorbates (presumably atomic oxygen) along lattice axes which are energetically favorable for the adsorbate-covered surface, while less so for the bare copper. In the deaerated electrolyte the stabilization through the adsorbate is missing and electrochemical annealing results in rounded surface shapes. This is in line with different step edge orientations observed for Cu(100) in the deaerated electrolyte at more cathodic potentials. Under these conditions, no oxygen should be adsorbed, in fact, every adsorbate except for possibly hydrogen and CO will have desorbed. This results in crystallographically-oriented step edges aligned with the Cu<110> axes as expected for a bare copper surface (Figure S6c).

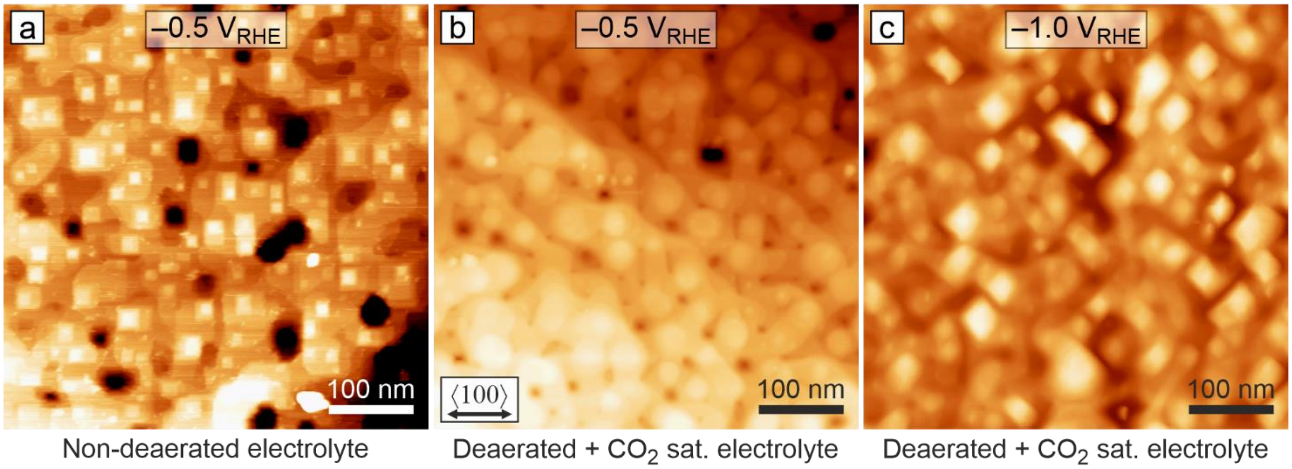


**Figure S6.** Evidence of specific adsorption on surface morphology. Comparison of *in situ* EC-AFM images of electropolished Cu(100) surfaces after reduction in non-deaerated and $CO_2$-saturated 0.1 M $KHCO_3$ recorded at different electrochemical reduction potentials: –0.5 $V_{RHE}$ and –1.0 $V_{RHE}$. Image sizes 500 nm × 500 nm.

## 8. Cu(100) in argon saturated 0.1 M $KHCO_3$

In order to assess possible electrolyte influences on surface morphologies EC-AFM images recorded in $CO_2$-free Ar-saturated electrolyte are shown in Figure S7 for comparison. Morphology imaged at OCV and –1.0 $V_{RHE}$ in Ar-saturated 0.1 M $KHCO_3$ strongly resembles that seen in $CO_2$-saturated electrolyte, but (b) shows a reduced smoothing into curved island shapes at –0.5 $V_{RHE}$. This hints at an effect of $CO_2$ or $CO_2$RR intermediates onto the electrochemical annealing. Image sizes: 400 nm × 200 nm. Figure S7d shows the comparison of cyclic voltammograms (CVs) in $CO_2$ vs. Ar- saturated electrolyte. Observable differences are an overall shift of the CV in Ar-electrolyte explained by higher pH in this electrolyte.

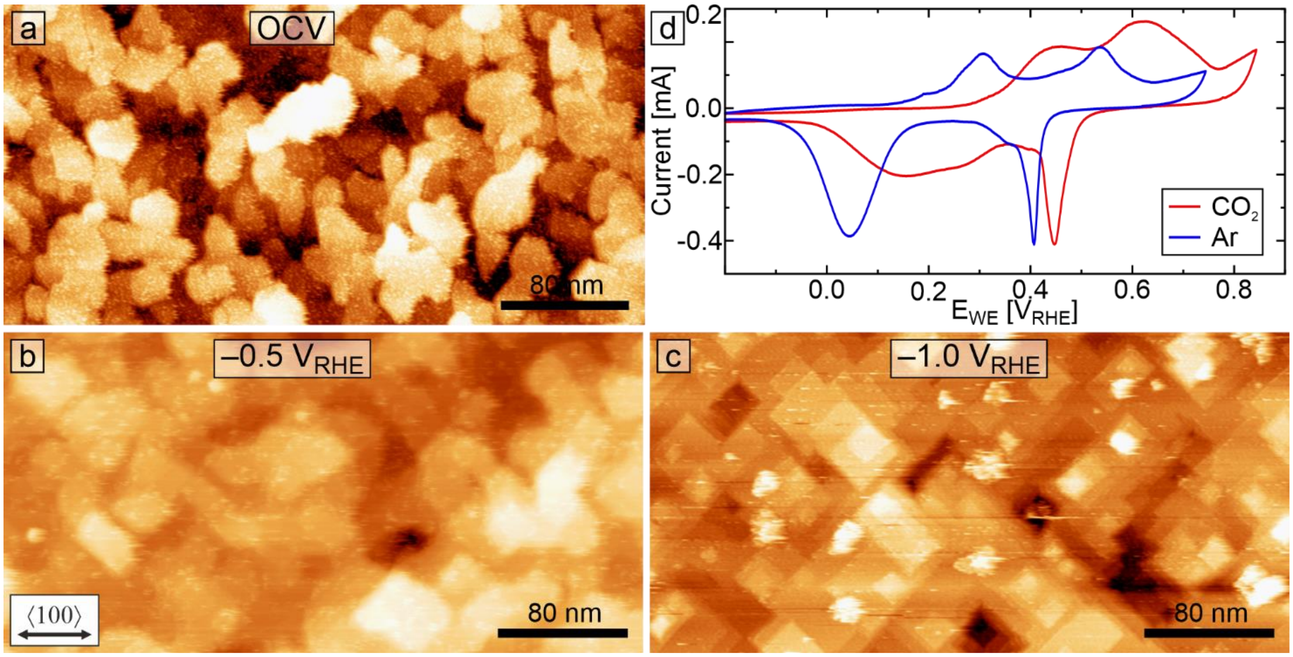


**Figure S7:** Argon saturated 0.1 M $KHCO_3$ electrolyte. (a-c) *In situ* EC-AFM images of a Cu(100) electrode at OCV, –0.5 $V_{RHE}$ and –1.0 $V_{RHE}$ for comparison with images in Figures of the main text recorded in $CO_2$-saturated electrolyte. (d) CV of a Cu(100) electrode in Ar-saturated 0.1 M $KHCO_3$ electrolyte compared to one recorded in $CO_2$-saturated solution. Scan rates: 50 mV/s.

## 9. Adsorbates on Cu(100)

| Adsorbate | Reconstruction | Environment | Reference |
|---|---|---|---|
| **CO** | c(2x2), **p(2x2)** | UHV | [4] |
| O, O+CO | (√2x√2)R45°, (√2x2√2)R45° | UHV | [5] |
| CO | c(2x2) | UHV | [6] |
| Cl+$H_2O$ | c(2x2) | UHV | [7] |
| Li+CO | c(2x2)-Li | UHV | [8] |
| N | c(2x2) | UHV | [9] |
| I | **p(2x2)-I** | UHV | [10] |
| **Te** | **p(2x2)-Te** | UHV | [11] |
| **Pd** | **p(2x2)-Pd,** (2 × 2)p4g-Pd | UHV | [12] |
| **S** | **p(2x2)-S** | UHV | [13] |
| **S** | **p(2x2)-S** | UHV | [14] |
| Pb | c(4x4), c(2x2), c(5√2x√2)R45° | UHV | [15] |
| Pb+Cl | c(2x2) | electrolyte | [16] |
| CO | c(2x2) | electrolyte | [17] |
| **S+halide** | **p(2x2)-S**, c(6x2) | electrolyte | [18] |
| Cl, Br | c(2x2) | electrolyte | [18] |

**Table S1.** Some literature references on adsorbate structures on Cu(100) from UHV and electrolyte studies with emphasis on the common c(2x2) and the less common p(2x2) reconstructions.

## 10. Gas evolution in the EC-AFM cell during imaging

Gas evolution within the EC-cell is a major problem for most, if not all *in situ* / *operando* characterization methods. Gas bubbles (i) alter the electrode-electrolyte interfacial properties, (ii) those of the electrolyte or (iii) simply obscure the electrode-liquid interface. In EC-AFM all three aspects are relevant. Confinement of the educt-depleted electrolyte may alter the double layer region and the presence of gas may change the local electrolyte composition (e.g local pH). Additionally, gas bubbles can block the optical paths of the AFM excitation and detection lasers which prevents imaging during the passage or residence time of the bubble. Figure S8a shows an optical micrograph of the EC-AFM cell during imaging in the presence of a bubble emerging from underneath the cantilever chip, which eventually altered the laser path sufficiently to prevent detection. Figure S8b shows the case of small stationary bubbles in the vicinity of the cantilever which do not interfere with the detection and would allow imaging. In certain cases of continuous gas evolution, imaging precedes despite bubbles emerging near the cantilever. The temporarily blocked optical path, resulting in image segments without signal, is visible as stripes with no contrast parallel to the fast scan direction (x- or horizontal axis), see Figure S4b. Sometimes imaging resumes after a bubble perturbance without visible effect, other times the piezo creep leads to artifacts in the image.

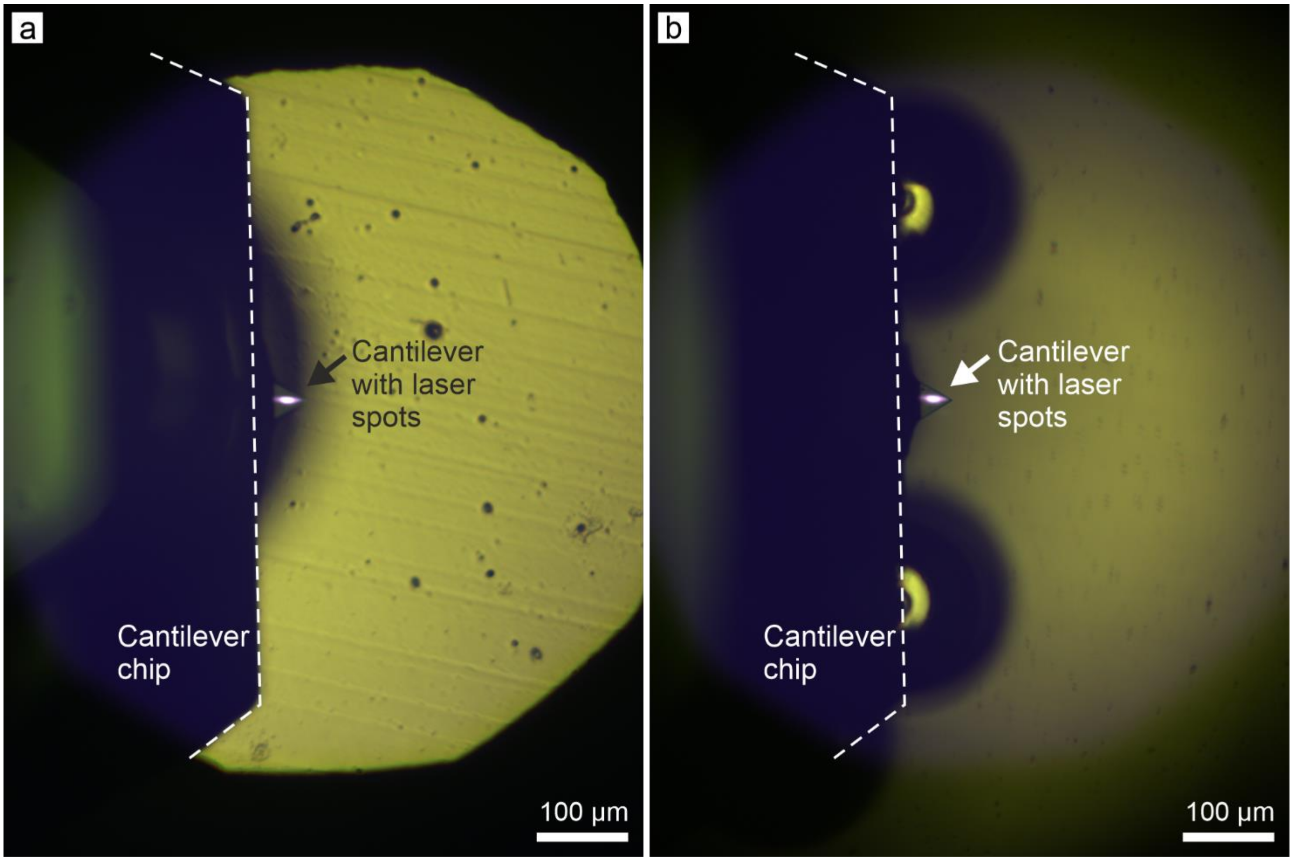


**Figure S8.** Gas bubbles in the EC-AFM cell. (a) Gas bubble emerging from underneath the cantilever chip and close to blocking the optical path of the laser. (b) Two small stationary bubbles near the cantilever, which would not prohibit imaging, but may have an effect on the sample by further confining the electrolyte volume and changing local gradients.

## Supplementary references


1. Fukuma, T., *Wideband low-noise optical beam deflection sensor with photothermal excitation for liquid-environment atomic force microscopy.* 2009. **80**(2): p. 023707.
2. Lampimäki, M., et al., *Nanoscale oxidation of Cu(100): Oxide morphology and surface reactivity.* 2007. **126**(3): p. 034703.
3. Vogt, M.R., et al., *Adsorbate-induced step faceting of Cu(100) electrodes in HCl.* Surface Science, 1996. **367**(2): p. L33-L41.
4. Joyner, R.W., C.S. McKee, and M.W. Roberts, *The adsorption of carbon monoxide on Cu(001): LEED and Auger emission studies.* Surface Science, 1971. **26**(1): p. 303-309.
5. Habraken, F.H.P.M., C.M.A.M. Mesters, and G.A. Bootsma, *The adsorption and incorporation of oxygen on Cu(100) and its reaction with carbon monoxide; comparison with Cu(111) and Cu(110).* Surface Science, 1980. **97**(1): p. 264-282.
6. Yu, H., D.Q. Hu, and K.T. Leung, *Electron energy loss studies of room-temperature co adsorption on Cu(100) induced by electron or ion irradiation.* Progress in Surface Science, 1995. **50**(1): p. 197-205.
7. Puisto, M., et al., *LEED – IV and DFT study of the co-adsorption of chlorine and water on Cu(100).* Surface Science, 2017. **657**: p. 51-57.
8. Tero, R., T. Sasaki, and Y. Iwasawa, *CO Adsorption on c(2×2)-Li/Cu(100): interaction between CO and Li on unreconstructed Cu(100) surfaces.* Surface Science, 2000. **448**(2): p. 250-260.
9. Leibsle, F.M., et al., *STM observations of Cu(100)−c(2×2)N surfaces: evidence for attractive interactions and an incommensurate c(2 × 2) structure.* Surface Science, 1994. **317**(3): p. 309-320.
10. Citrin, P.H., P. Eisenberger, and R.C. Hewitt, *Adsorption Sites and Bond Lengths of Iodine on Cu{111} and Cu{100} from Surface Extended X-Ray-Absorption Fine Structure.* Physical Review Letters, 1980. **45**(24): p. 1948-1951.
11. Comin, F., et al., *Unusual chemisorption behavior of Te on Cu{111} versus Cu {100}.* Physical Review B, 1982. **26**(12): p. 7060-7062.
12. Shen, Y.G., et al., *Reinvestigation of the surface reconstruction of Cu(001)-(2 × 2)p4g-Pd.* Surface Science, 1997. **394**(1): p. L131-L137.
13. Vlieg, E., I.K. Robinson, and R. McGrath, *Structure determination of Cu(100)-p(2 × 2)-S using x-ray diffraction.* Physical Review B, 1990. **41**(11): p. 7896-7898.
14. Walen, H., et al., *Sulfur Atoms Adsorbed on Cu(100) at Low Coverage: Characterization and Stability against Complexation.* The Journal of Physical Chemistry B, 2018. **122**(2): p. 963-971.
15. Nagl, C., et al., *Surface alloying and superstructures of Pb on Cu(100).* Surface Science, 1995. **331-333**: p. 831-837.
16. Wu, H.-C. and S.-L. Yau, *In Situ Scanning Tunneling Microscopy of Underpotential Deposition of Lead on Cu(100) in Sulfuric Acid Solutions.* The Journal of Physical Chemistry B, 2001. **105**(29): p. 6965-6971.
17. Baricuatro, J.H., et al., *Seriatim ECSTM-ECPMIRS of the adsorption of carbon monoxide on Cu(100) in alkaline solution at CO2-reduction potentials.* Electrochemistry Communications, 2018. **91**: p. 1-4.
18. Rahn, B. and O.M. Magnussen, *Sulfide Surface Dynamics on Cu(100) and Ag(100) Electrodes in the Presence of c(2×2) Halide Adlayers.* 2018. **5**(20): p. 3073-3082.
